# Metabolic and Structural Signatures of Speech and Language Impairment in Corticobasal Syndrome: A Multimodal PET/MRI Study

**DOI:** 10.3389/fneur.2021.702052

**Published:** 2021-08-30

**Authors:** Jacy Bezerra Parmera, Isabel Junqueira de Almeida, Marcos Castello Barbosa de Oliveira, Marcela Lima Silagi, Camila de Godoi Carneiro, Adalberto Studart-Neto, Carla Rachel Ono, Egberto Reis Barbosa, Ricardo Nitrini, Carlos Alberto Buchpiguel, Sonia Maria Dozzi Brucki, Artur Martins Coutinho

**Affiliations:** ^1^Department of Neurology, Hospital das Clínicas, Faculdade de Medicina da Universidade de São Paulo, São Paulo, Brazil; ^2^Department of Physical Therapy, Speech, and Occupational Therapy, Hospital das Clínicas, Faculdade de Medicina da Universidade de São Paulo, São Paulo, Brazil; ^3^Neurology Unit, Instituto do Câncer do Estado de São Paulo, São Paulo, Brazil; ^4^Department of Speech, Language and Hearing Sciences, Universidade Federal de São Paulo, São Paulo, Brazil; ^5^Laboratory of Nuclear Medicine, Nuclear Medicine Center and Division, Hospital das Clínicas, Faculdade de Medicina da Universidade de São Paulo, São Paulo, Brazil

**Keywords:** corticobasal syndrome, frontotemporal lobar degeneration, nonfluent primary progressive aphasia, positron emission tomography, amyloid-PET, fluorodeoxyglucose F18, corticobasal degeneration

## Abstract

**Introduction:** Corticobasal syndrome (CBS) is a progressive neurological disorder related to multiple underlying pathologies, including four-repeat tauopathies, such as corticobasal degeneration and progressive supranuclear palsy, and Alzheimer's disease (AD). Speech and language are commonly impaired, encompassing a broad spectrum of deficits. We aimed to investigate CBS speech and language impairment patterns in light of a multimodal imaging approach.

**Materials and Methods:** Thirty-one patients with probable CBS were prospectively evaluated concerning their speech–language, cognitive, and motor profiles. They underwent positron emission tomography with [^18^F]fluorodeoxyglucose (FDG-PET) and [^11^C]Pittsburgh Compound-B (PIB-PET) on a hybrid PET-MRI machine to assess their amyloid status. PIB-PET images were classified based on visual and semi-quantitative analyses. Quantitative group analyses were performed on FDG-PET data, and atrophy patterns on MRI were investigated using voxel-based morphometry (VBM). Thirty healthy participants were recruited as imaging controls.

**Results:** Aphasia was the second most prominent cognitive impairment, presented in 67.7% of the cases, following apraxia (96.8%). We identified a wide linguistic profile, ranging from nonfluent variant-primary progressive aphasia to lexical–semantic deficits, mostly with impaired verbal fluency. PIB-PET was classified as negative (CBS-A– group) in 18/31 (58%) and positive (CBS-A+ group) in 13/31 (42%) patients. The frequency of dysarthria was significantly higher in the CBS-A– group than in the CBS-A+ group (55.6 vs. 7.7%, *p* = 0.008). CBS patients with dysarthria had a left-sided hypometabolism at frontal regions, with a major cluster at the left inferior frontal gyrus and premotor cortex. They showed brain atrophy mainly at the opercular frontal gyrus and putamen. There was a positive correlation between [^18^F]FDG uptake and semantic verbal fluency at the left inferior (*p* = 0.006, *R*^2^ = 0.2326), middle (0.0054, *R*^2^ = 0.2376), and superior temporal gyri (*p* = 0.0066, *R*^2^ = 0.2276). Relative to the phonemic verbal fluency, we found a positive correlation at the left frontal opercular gyrus (*p* = 0.0003, *R*^2^ = 0.3685), the inferior (*p* = 0.0004, *R*^2^ = 0.3537), and the middle temporal gyri (*p* = 0.0001, *R*^2^ = 0.3993).

**Discussion:** In the spectrum of language impairment profile, dysarthria might be helpful to distinguish CBS patients not related to AD. Metabolic and structural signatures depicted from this feature provide further insights into the motor speech production network and are also helpful to differentiate CBS variants.

## Introduction

Corticobasal syndrome (CBS) is a rare progressive neurological disorder distinguished by asymmetric motor features and higher cortical dysfunction associated with general cognitive impairment (1). Initially described as a clinicopathological entity (2), it is now considered a clinical phenotype related to multiple underlying pathologies (3). The majority of cases are due to four-repeat (4R) tauopathies (4), mainly corticobasal degeneration (CBD) (5), followed by progressive supranuclear palsy (PSP) (6, 7). Also, possible underlying pathologies include Alzheimer's disease (AD) (8, 9) and frontotemporal lobar degeneration with transactivation response (TAR) DNA binding protein 43 kDa (TDP-43) inclusions (7), among others (10–12).

Besides motor symptoms, cognitive and behavioral disturbances are common and often recognized as the first presentation in CBS (13, 14). Additionally, prominent language dysfunction is usually present from the early stages or during the disease course (1, 15, 16) and incorporated into previous diagnostic criteria (17).

Previous studies assessing the broad spectrum of speech and language in CBS patients have reported a phenotype similar to the nonfluent variant of primary progressive aphasia (nfv-PPA) and the primary progressive apraxia of speech (PPAOS) (18). Individuals may fulfill the criteria for nfv-PPA (19) or PPAOS (20) and only, later on, fit into probable CBS criteria (21, 22). Moreover, other studies described a wide variety of language deficits: Broca's aphasia, anomic aphasia, and fluent aphasia (23).

Recently, studies using imaging biomarkers such as structural magnetic resonance (MRI) (24), [^18^F]fluorodeoxyglucose (FDG)-positron emission tomography (FDG-PET) (25), and amyloid-PET (26) identified neural correlates from different aspects of language in CBS. Nevertheless, language impairment's profile in CBS and its relation to specific pathologies are still poorly understood.

This study aimed to investigate language and motor speech impairment in CBS patients in light of a multimodal imaging approach. Our main purpose was to compare speech–language deficits in CBS patients related to the presence or absence of brain amyloid deposition on amyloid-PET, a surrogate for underlying AD pathology. We also intended to explore metabolic and structural signatures related to these speech–language profiles.

## Materials and Methods

### Participants

Thirty-one patients meeting the probable CBS (1) criteria were prospectively recruited at the movement disorders and cognitive neurology clinics at Hospital das Clínicas, University of São Paulo School of Medicine (São Paulo, Brazil), between February 2017 and December 2019. First, they were classified by assistant doctors (all board-certified neurologists) at both clinics as having probable CBS. Later, all individuals were further evaluated regarding their clinical profile to perform the study protocol by two neurologists (JBP and SMDB) with board certification in both movement disorders and cognitive neurology. All patients showed a progressive disease course with a duration of at least 1.5 years. They also presented an asymmetric combination of at least two out of three motor features, including akinetic-rigid parkinsonism, dystonia, and myoclonic movements, as well as two out of three higher cortical features, including limb or orobuccal apraxia, alien limb phenomena, and cortical sensory deficit (1). Then, alternative diagnoses among neurodegenerative diseases could be excluded, such as Creutzfeldt-Jakob disease, other atypical parkinsonian syndromes, Parkinson's disease, typical AD, and others.

Exclusion criteria included relevant non-degenerative brain lesions such as stroke sequelae, tumors, hydrocephalus, and remarkable premorbid psychiatric disease. All participants or their caregivers provided written informed consent for the study. The ethical committee of our institution approved the investigation procedure and informed consent under protocol number 2.046.113.

We also included 30 cognitively healthy participants (NC group) from the community as imaging controls after neuropsychological and neurological evaluations. They were all participants of another prospective research of our group (under protocol number 62047616.0.0000.0068). They matched the CBS patients by age (median age 67.0, interquartile range [IQR] 62.25–70.0) and scanner type. Data concerning demography and neuropsychological evaluation obtained from the healthy controls are available in Supplementary Table 1.1.

### Clinical Assessment

All patients received a standardized predefined clinical evaluation. Global cognitive impairment was assessed with Addenbrooke's Cognitive Examination-Revised (ACER) (27–29) and the Mini-Mental State Examination (MMSE) (30), both previously validated in Brazilian cohorts. Episodic memory was investigated with the Brief Cognitive Screening Battery (BCSB) (31), a test used to assess individuals with different educational backgrounds and attention or working memory with the backward digit span. Functional decline was assessed with the Clinical Dementia Rating scale (32) and Functional Activities Questionnaire (33).

Higher cortical functions were clinically evaluated by the presence of limb or orobuccal apraxia, cortical sensory deficits, alien limb phenomena, and Balint and Gerstmann syndromes. We characterized the presence of limb apraxia by imitation of meaningful and meaningless gestures and with imaginary tool use and orobuccal apraxia by meaningless orobuccal gestures (34).

A detailed examination of the motor signs was performed through a neurological examination that characterized the presence of parkinsonism, dystonia, and myoclonus. The motor impairment was also categorized by the Hoehn and Yahr scale (35).

The neurologists also questioned the participants and caregivers about their first symptoms and, together with major signs at first examination, designated the predominant clinical initial phenotype as mainly cognitive, motor, or language impairment. The extended motor and cognitive clinical assessment were described in a previous publication (36).

### Speech and Language Assessment

A comprehensive speech and language evaluation was performed by two speech–language pathologists (IJA and MLS), including the Western Aphasia Battery-revised (WAB-R) (37), the American Speech–Language–Hearing Association Functional Assessment of Communication Skills (ASHA-FACS) (38), and verbal fluency tests. From the WAB-R, the following subtests were utilized: spontaneous speech, verbal comprehension, repetition, naming, and word finding. The aphasia quotient (AQ), a measure of aphasia severity, was derived from those tests. ASHA-FACS is a scale that measures functional communication. It evaluates the level of assistance that the patient needs to communicate effectively.

We also evaluated the presence of apraxia of speech (AOS), agrammatism, and dysarthria. AOS was evaluated based on all the speech productions and complemented by the following tasks: oral diadochokinesis, repetition of polysyllables, multiple repetitions of the same polysyllable, repetition of words that increase in length by suffix and prefix derivation, repetition of dissyllables, and dissociation between voluntary and automatic production. The presence of agrammatism was judged based on all oral productions and, when available, written productions.

Dysarthria was characterized as present or absent considering the different manifestations in the motor speech bases (i.e., breathing, phonation, articulation, resonance, and prosody), through the evaluation of reflexes (coughing and swallowing), saliva control, breathing, tonus, and mobility of phonoarticulatory structures (tongue, lips, jaw, palatine veil, and larynx), and speech intelligibility.

To characterize the presence of aphasia, we compared the AQ score of each CBS patient to the median value of the AQ of other 24 healthy control subjects with the same age and education level. If these data were not available, we categorized aphasia based on the language score at ACE-R with a cutoff obtained from a previous Brazilian study, based on age and formal education (29).

For the semantic fluency task, participants were asked to name as many animals as possible in 1 min. Participants named words beginning specifically with the letter P for the phonemic fluency task, which was assessed using the ACE-R. Based on a previous survey of a Brazilian sample, we determined cutoff scores of 9 for semantic fluency for illiterates or individuals with <8 years of formal education and 13 for persons with more than 8 years of formal education (39). We determined cutoff scores of 13 for phonemic fluency for illiterates or individuals with <8 years of education and 15 for persons with more than 8 years of education (40).

### Neuroimaging Data Acquisition

Both [^11^C]Pittsburgh Compound-B (PIB) and [^18^F]FDG were produced in an on-site cyclotron (PET trace 880, GE Healthcare) at the Nuclear Medicine Center of the Institute of Radiology (CMN InRad, São Paulo, SP, Brazil) of our hospital. PIB-PET and MRI images were simultaneously acquired on a hybrid 3.0-T SIGNA PET/MRI scanner (GE Healthcare, Milwaukee, WI). The MRI protocol included volumetric sequences weighted on T1, T2, and T2/FLAIR (fluid attenuation inversion recovery) sequences, as well as diffusion-weighted imaging (DWI) in 6 and 33 directions, and susceptibility-weighted imaging (SWI). All images were visually inspected for the detection of structural lesions of the brain, skull, and head and neck lesions, as well as for the assessment of imaging artifacts that could impair imaging processing. Complete parameters of the MRI sequences are detailed as follows: T1-weighted (spoiled gradient recalled, SPGR), TR = 8 ms, TE = 3 ms, FOV (cm) = 25.6, slice thickness = isometric voxels of 1.0 × 1.0 × 1.0 mm, frequency = 256, phase = 256, NEX = 1, scan time = 5 min 16 s, [TI] = 600 ms, flip angle [FA] = 8, r, 196 sagittal slices; T2-weighted (CUBE technique), TR = 2,500 ms, TE = 88 ms, FOV (cm) = 25,6, slice thickness = isometric voxels of 1.0 × 1.0 × 1.0 mm, frequency = 256, phase = 256, NEX = 1, scan time (min) = 3 min 43 s, [TI] = 600 ms, flip angle [FA] = 90, r, 196 sagittal slices; FLAIR, TR = 6,500 ms, TE = 141 ms, FOV (cm) = 25.6, slice thickness (mm) = isometric voxels of 1.3 × 1.3 × 1.3 mm, frequency = 192, phase = 192, NEX = 1, scan time (min) = 4 min 4 s, [TI] = 1,905 ms, flip angle [FA] = 90, r, 152 sagittal slices; DTI 33 dir and DTI 6 dir, TR (ms) = 1,300 ms, TE (ms) = 73.9, FOV (cm) = 25.6, slice thickness (mm) = 2.2 × 2.2 × 2.2 mm, frequency = 116, phase = 116, NEX = 1, scan time (min) = 9 min 32 s and 2 min 36 s, [TI] = 1,905 ms, flip angle [FA] = 90, r, 152 sagittal slices *b*-value 1,000, 33 directions; T2 images, 10 and 6 directions, no of T2 images = 5; Ax SWAN QSM, TR (ms) = minimum, TE = 29 ms, FOV (cm) = 24, slice thickness (mm) = 2, frequency = 480, phase = 480, NEX = 1, scan time (min) = 13 min 37 s, [TI] = 1,905 ms, flip angle [FA] = 90, r, 152 sagittal slices.

FDG-PET was acquired in a Discovery 710 PET/CT scanner (GE Healthcare, Milwaukee, WI). The radiotracer [^18^F]FDG was injected intravenously in bolus with a mean activity of 5–6 mCi. Before the radiopharmaceutical injection of FDG, the subjects fasted for at least 6 h, and their blood glucose level was <180 mg/dl. The time interval between injection and scan start was at least 30 min, and scan duration was 15 min. Each PET scan was corrected for attenuation with CT data. Images were reconstructed using an ordered subset expectation maximization (OSEM) algorithm.

The production of the radiopharmaceutical compound PIB was entirely carried out in the cyclotron of our center and previously validated in our environment (41). The images of cortical amyloid deposition were analyzed in the acquisition time of 30 min, obtained in rest conditions, between 40 and 70 min after intravenous administration of 10–15 mCi of the radiopharmaceutical.

The FDG-PET was performed within 1 month after clinical examination, and the time between FDG and PIB-PET/MRI varied from 2 days to 6 months.

### [^11^C]PIB-PET Visual Classification

Two nuclear medicine physicians performed a visual evaluation of the PIB-PET images assisted by a 3D-SSP semi-quantitative software (Cortex ID Suite, GE healthcare). Participants were rated as “CBS-A+” or “CBS-A–” if they were positive or negative, respectively, for the presence of cortical amyloid deposition, according to previously established criteria (42). A previous study from our group observed a high interrater agreement and similar amyloid positivity rates from the literature (43).

### Quantitative [^18^F]FDG-PET Analysis

Quantitative FDG-PET group analyses were performed to investigate (1) which brain areas were more consistently hypometabolic in CBS patients compared to healthy controls; (2) which were the most consistently hypometabolic areas in CBS patients concerning the difference in language performance; and (3) which brain areas were correlated to the scores on phonemic and semantic verbal fluency tests.

PET images were co-registered with their respective MRI images (volumetric T1 sequence) and spatially normalized using the Statistical Parametric Mapping 8 (SPM8) software (Wellcome Department of Cognitive Neurology, Functional Imaging Laboratory, London, UK) into an anatomic template (44). To perform the first investigation mentioned above, we flipped the images to represent the hemisphere contralateral to the most affected limbs on the right side of the image because of CBS's asymmetric nature. The second and third analyses were performed within the images in their original lateralization to evaluate aspects of language hemisphere dominance.

The spatial normalization of FDG-PET scans was performed using a dementia-optimized brain FDG-PET template (44). Scans were smoothed with an 8-mm full width at half maximum Gaussian kernel to reduce misregistration into the template space and improve the signal-to-noise ratio. A default threshold of 0.8 of the mean uptake inside the brain was selected to ensure that the analysis included only voxels mapping cerebral tissue. Global uptake differences were adjusted using the “proportional scaling” SPM8 option.

For the group analyses, statistical parametric maps were generated with SPM8 threshold at the voxel level at *p* uncorrected (punc) = 0.001, with a minimum extension of 100 voxels in the cluster. Statistical results were considered valid when they survived correction for multiple comparisons with the familywise error (FWE) or false discovery rate (FDR) methods (pFWE/FDR ≤ 0.05). Relevant peak voxels from the statistical parametric maps were identified in the Montreal Neurologic Institute (MNI) coordinate system.

The numeric values representing the mean [^18^F]FDG uptake for each individual (a proxy for regional brain glucose metabolism, rBGM) in the clusters with statistically significant results in the SPM group analyses) were obtained with the toolbox MarsBar for SPM (http://marsbar.sourceforge.net/) and later investigated using GraphPad Prism version 6.0 (GraphPad Software, La Jolla, CA, USA).

### Voxel-Based Morphometry Analysis

We performed quantitative voxel-based MRI group analyses to investigate (1) brain atrophy patterns in CBS patients compared to healthy controls and (2) brain atrophy patterns in CBS patients in relation to the difference in language performance compared to healthy controls.

Like in the FDG-PET quantitative analysis, we flipped the images to represent the hemisphere contralateral to the most affected limbs on the right side in the first step of the investigation. The second analysis was performed within the images in their original lateralization to evaluate language hemisphere dominance aspects.

MRI T1-weighted volumetric images were processed using VBM on SPM8 using the SPM toolbox *Diffeomorphic Anatomical Registration using Exponentiated Lie algebra* (DARTEL) algorithm. This algorithm segmented MRI images into liquor, gray matter, and white matter.

### Study Design

First, the patients were prospectively selected and clinically assessed (sections Participants, Clinical Assessment, and Speech and Language Assessment). They underwent FDG-PET, MRI, and PIB-PET and were classified as CBS-A– and CBS-A+, according to the PIB-PET status (described in section [^11^1C]PIB-PET visual classification). After this initial distribution, both groups were compared concerning the clinical evaluation and speech and language assessment, aiming to possibly delineate the different clinical variants based on the presence of cortical amyloid deposition. Later, we performed quantitative group analyses to compare brain metabolic patterns and brain atrophy patterns between the whole CBS group and healthy controls and between CBS patients concerning differences in language performances and healthy controls.

### Statistical Analysis of Clinical Data

Demographic, clinical, and language data analysis was conducted in R (https://www.r-project.org/). Categorical variables were expressed as absolute and relative frequencies and compared with Pearson's chi-square (or Fisher's exact test, as appropriate). Continuous variables were compared using the Mann–Whitney test after failing to satisfy normality through visual inspection of their distribution. Data were expressed as median [IQR] or as number [frequency]. All tests were two-sided. Statistical significance was set as *p* < 0.05.

## Results

### Demography and Clinical Features

Thirty-one CBS patients were included and underwent a comprehensive clinical evaluation. Demographic data are shown in Table 1. Eighteen patients presented initially with a cognitive clinical phenotype (58.1%), followed by 10 patients with motor (32.3%) and 3 (9.7%) with a predominant language profile (Table 1). These three patients possibly could have shown an nfv-PPA phenotype, based on chart review or patient report, and then evolved into probable CBS before enrollment in the study.

**Table 1:** Demography, functional, cognitive, and language assessment of patients with CBS and comparison by amyloid-PET results.

	**CBS (*n* = 31)**	**CBS-A– (*n* = 18)**	**CBS-A+ (*n* = 13)**	***p*-value**
**Demography**
Age at symptom onset, years	61 (58–67)	60 (55–68)	63 (60–66)	ns
Age at main assessment, years	65 (61–71)	63.5 (59–71)	66 (64–71)	ns
Symptom duration at main assessment, years	4.0 (3.0–4.5)	3.5 (2.2–4.7)	4.0 (3.0–4.0)	ns
Gender (female)	14 (45.2%)	7 (38.9%)	7 (53.8%)	ns
Education, years	10 (6–15)	9.5 (6–15)	10 (6–15)	ns
Side of more severely involved limbs (right)	13 (41.9)	8 (44.4%)	5 (38.5%)	ns
Handedness (right-handed)	26 (83.9%)	16 (88.9%)	10 (76.9%)	ns
Phenotype
Cognitive	18 (58.1%)	8 (44.4%)	10 (76.9%)	
Motor	10 (32.3%)	7 (38.9%)	3 (23.1%)	
Language	3 (9.7%)	3 (16.7%)	0 (0.0%)	ns
**Functional assessment**
Clinical Dementia Rating	2.0 (1.5–2.0)	2.0 (0.6–2.0)	2.0 (1.0–2.0)	ns
Functional activities questionnaire	22 (14–26)	18.5 (11–25)	25 (16–27)	ns
Hoehn and Yahr scale	2 (2–3.5)	3.00 (2–3.75)	2.00 (2–3)	ns
ASHA-FACS scale	3.2 (1.8–5.3)	3.2 (2.4–5.0)	3.0 (1.6–5.0)	ns
**General cognitive assessment**
ACE-R total	41 (30–62)	49 (31.5–74.5)	34 (27.5–46.5)	ns
ACE-R attention	11 (9–13.75)	12.5 (11–16.25)	9 (8–10.5)	0.008
ACE-R memory	8 (5.25–15.75)	12.5 (7.75–18.25)	5 (2.25–8)	0.008
ACE-R fluency	2.5 (1–6)	3 (2–6.25)	1.5 (1–4.5)	ns
ACE-R language	16.5 (14–24.5)	19 (14.25–25)	14.5 (14–20.75)	ns
ACE-R visuospatial	7 (4–8.75)	8 (7–11.25)	4 (3.25–5.75)	0.001
MMSE	18 (13–21.50)	20.5 (16.5–25.75)	14 (11–17)	0.005
Digits backward	2 (0–3.75)	3 (2–3)	0 (0–4)	ns
Delayed recall (BCSB)	3 (0.5–6)	5.50 (1.75–6)	1 (0–3)	ns
**Language assessment**
Aphasia quotient (WAB-R)	68.8 (51.1–88.2)	70.35 (38.7–83.3)	68.8 (63.7–90.2)	ns
Total spontaneous speech (WAB-R)	16.0 (9.5–17.5)	17.0 (10.0–18.0)	14.5 (10.0–16.75)	ns
Auditory word recognition (WAB-R)	54.0 (19.0–57.5)	57.0 (48.0–60.0)	50.0 (25.0–55.0)	ns
Sequential commands (WAB-R)	63.0 (25.0–80.0)	63.0 (28.0–80.0)	48.0 (15.2–73.2)	ns
Total repetition (WAB-R)	8.6 (3.3–9.1)	8.6 (3.8–9.2)	7.6 (3.0–8.9)	ns
Naming and word finding (WAB-R)	6.2 (3.25–8.45)	7.1 (3.3–8.5)	5.4 (2.5–7.1)	ns
Phonemic fluency (letter P)	3 (1.75–6)	3 (2–6.25)	2.5 (1–5.25)	ns
Semantic fluency (animals)	5.5 (3.75–10)	6.5 (3–11.75)	5 (4–7)	ns

*Clinical data comparison between CBS-A+ and CBS-A–. Data expressed as median (IQR) or number (frequency). Statistical significance was set as p < 0.05 (Mann–Whitney or Fischer's exact test). ns, nonsignificant; AD, Alzheimer's disease; CBS, corticobasal syndrome; MMSE, Mini-mental State Examination; ACE-R, Addenbrooke Cognitive Examination-Revised; BCSB, Brief Cognitive Screening Battery; ASHA-FACS, Functional assessment of Communication Skills for Adults*.

**Figure 1:**
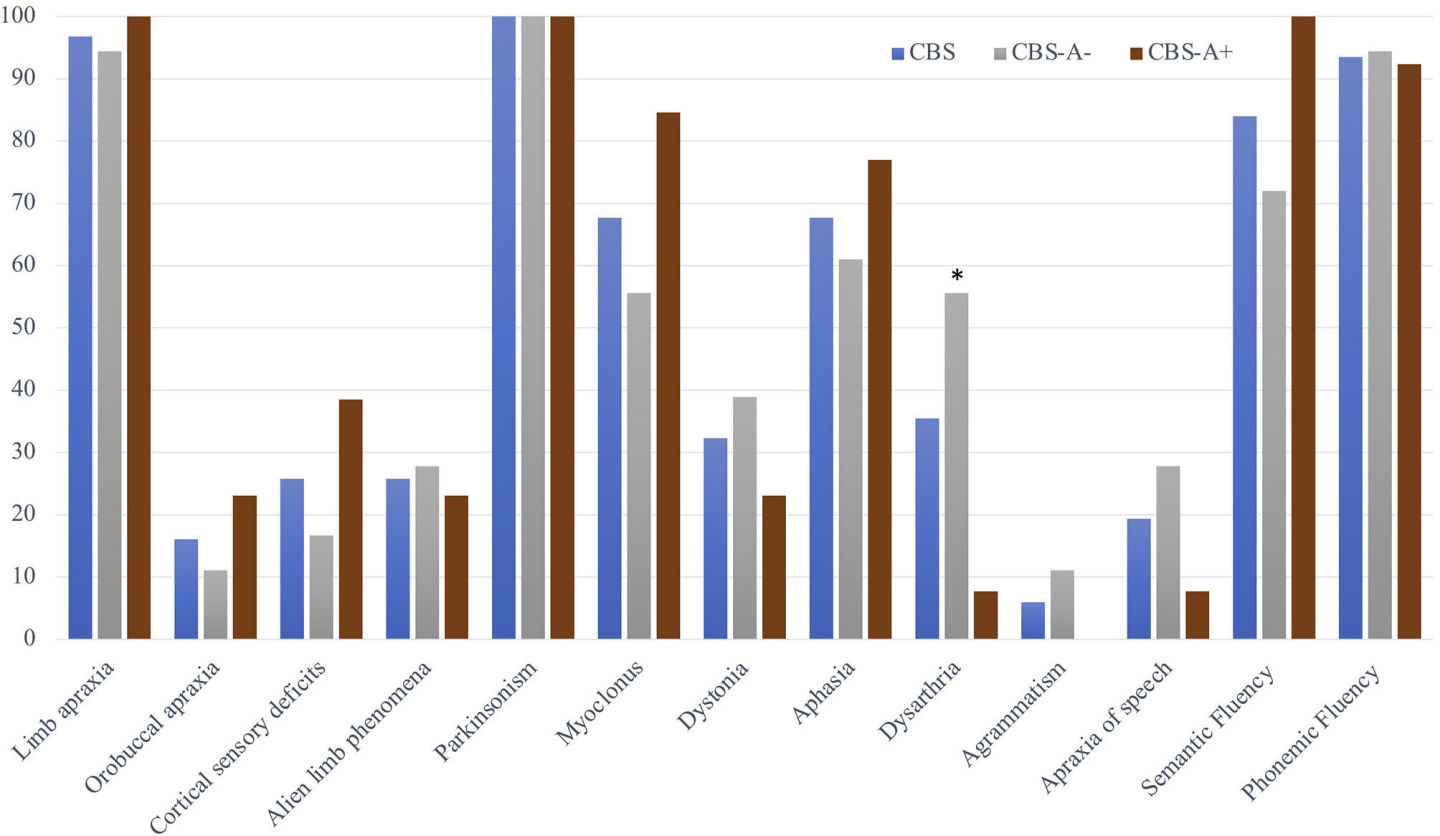
Motor, cortical, and language deficits in the whole CBS cohort and according to subgroups with positive (CBS-A+) and negative (CBS-A–) amyloid-PET. Data are presented as the frequency of the symptoms or the percentage of altered verbal fluency tasks among the CBS sample and in the subgroups according to cortical amyloid deposition. The symbol * indicates statistically significant differences between CBS-A+ and CBS-A– groups. Dysarthria 10/18, 55.6% vs. 1/13, 7.7%, *p* = 0.008, Fisher's exact test.

The motor features included asymmetric akinetic-rigid parkinsonism in all cases (100%). Dystonia was present in 10 (32.3%) and myoclonus in 21 (67.7%) patients. Limb apraxia was the most frequent cognitive sign, demonstrated in 30 (96.8%) patients. Buccolingual apraxia was less common, found in only five (16.1%). Cortical sensory deficits and alien limb phenomena were both present in eight (25.8%) cases. Two patients (6.45%) had Balint and Gerstmann syndromes (Figure 1 and Table 2).

**Table 2:** Clinical symptoms and signs of patients with CBS and comparison by amyloid-PET results.

	**CBS (*n* = 31)**	**CBS-A– (*n* = 18)**	**CBS-A+ (*n* = 13)**	***p*-value**
**Cortical symptoms**
Limb apraxia	30 (96.8%)	17 (94.4%)	13 (100.0%)	ns
Orobuccal apraxia	5 (16.1%)	2 (11.1%)	3 (23.1%)	ns
Cortical sensory deficits	8 (25.8%)	3 (16.7%)	5 (38.5%)	ns
Alien limb phenomena	8 (25.8%)	5 (27.8%)	3 (23.1%)	ns
**Motor symptoms**
Parkinsonism	31 (100.0%)	18 (100.0%)	13 (100.0%)	ns
Myoclonus	21 (67.7%)	10 (55.6%)	11 (84.6%)	ns
Dystonia	10 (32.3%)	7 (38.9%)	3 (23.1%)	ns
**Language symptoms**
Aphasia	21 (67.7%)	11 (61.1%)	10 (76.9%)	ns
Dysarthria	11 (35.48%)	10 (55.6%)	1 (7.7%)	0.008
Agrammatism	2 (6.45%)	2 (11.1%)	0 (0.0%)	ns
Apraxia of speech	7 (22.6%)	5 (27.8%)	2 (15.4%)	ns
Abnormal semantic fluency	26 (83.9%)	13 (72.2%)	13 (100.0%)	ns
Abnormal phonemic fluency	29 (93.5%)	17 (94.4%)	12 (92.3%)	ns

*Comparison between amyloid-PET positive (CBS-A+) and negative (CBS-A–). Data expressed as number (frequency). Statistical significance was set as p < 0.05 (Fischer's exact test)*.

Concerning speech and language features, 21 patients (67.7%) had aphasia according to standard deviations of the AQ at WAB-R test or normative values on language subtest at ACE-R (Figure 1 and Table 2). Most measures obtained from WAB-R showed impairment in naming, sentence comprehension, and spontaneous speech (Table 1). Phonemic and semantic verbal fluency tests were below the normative values in 29 (93.5%) and 26 (84%) patients of the whole sample, respectively. Dysarthria was detected in 11 (35.5%) and AOS in 7 (19.4%). Two patients (6.45%) presented agrammatism (Figure 1 and Table 2).

### Language, Cognitive, and Motor Features According to Amyloid-PET Status

PIB-PET was classified as negative (CBS-A–) in 18/31 (58%) and positive in 13/31 (42%) patients after visual and semi-quantitative classification of amyloid deposition. Demographic variables did not differ between CBS-A– and CBS-A+ groups (Table 1).

The CBS-A+ group performed significantly worse on cognitive assessment through MMSE and some ACE-R subscores (attention, memory, and visuospatial) but did not differ in total ACE-R score (Table 1). CBS-A+ patients had worse BCSB delayed recall performance, although it did not reach statistical significance (Table 1). There were no significant differences in higher cortical or motor symptoms or signs between groups (Figure 1 and Table 2).

Concerning motor speech and language deficits, patients with negative amyloid deposition on PIB-PET displayed dysarthria significantly more often than did the CBS-A+ group (10/18, 55.6% vs. 1/13, 7.7%, *p* = 0.008, Fisher's exact) (Figure 1 and Table 2). The main characteristics were mixed hypokinetic and spastic dysarthria. There were no statistically significant differences in the frequency of aphasia (*p* = 0.452, Fisher's exact) (Table 2) and scores in the functional language assessment at ASHA-FACS between CBS-A- and CBS-A+ groups (*p* = 0.961, Mann–Whitney) (Table 1). Only patients classified as CBS-A– showed agrammatism (two patients). Also, CBS-A– patients had AOS more often than did CBS-A+ patients, although not statistically significant (*p* = 0.35). All patients with a predominant language phenotype had negative amyloid-PET status (Table 1).

Interestingly, CBS-A– patients appeared to show more compromised phonemic verbal fluency (17/18, 94.4%) than semantic fluency (13/18, 72%), although this did not reach statistical significance (*p* = 0.177, Fisher's exact). Conversely, all patients (13/13, 100%) of the CBS-A+ group showed impaired semantic verbal fluency, and phonemic verbal fluency was impaired in 92.3% (12/13) of patients.

### Metabolic Patterns on FDG-PET

**Figure 2:**
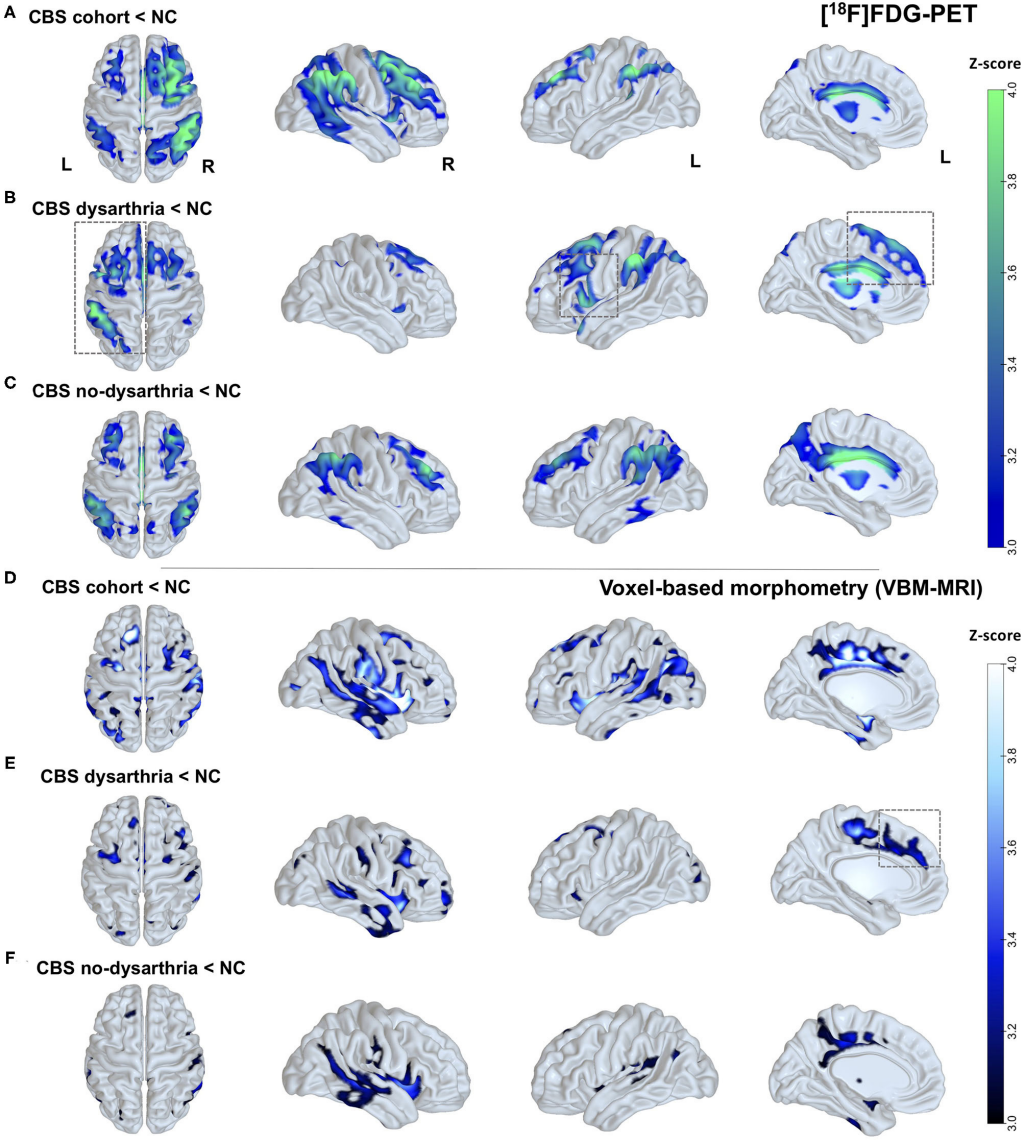
Brain glucose metabolism and brain atrophy patterns in patients with CBS and according to the presence or absence of dysarthria. **(A)** Clusters with differences in rBGM in individuals with CBS compared to healthy controls (NC). Reduced [^18^F]FDG uptake in the whole CBS cohort is consistently seen in the frontoparietal and temporal areas, striatum, and thalamus, mainly contralateral to the most affected side. **(B)** Clusters with differences in rBGM in CBS individuals presenting dysarthria. Reduced [^18^F]FDG uptake surviving correction for multiple comparisons at the cluster level is predominant at left frontal regions, with a major cluster at the left inferior frontal gyrus (opercular area) and left premotor cortex. **(C)** Hypometabolism in CBS patients without dysarthria showing bilateral rBGM reduction, mainly at the temporoparietal areas, striatum, and thalamus, and without hemisphere predominance. **(D)** VBM analysis showing brain atrophy patterns in CBS patients compared to NC: widespread brain atrophy pattern with major clusters at the bilateral striatum, SMA, and posterior temporoparietal areas, mostly contralateral to the affected body side. **(E)** VBM showing brain atrophy patterns in CBS patients with dysarthria compared to NC: predominantly in the frontal areas and striatum. **(F)** VBM showing brain atrophy patterns in CBS patients without dysarthria compared to NC: posterior temporal and inferior parietal areas. Parametric maps were generated with an unpaired *t*-test (*p* < 0.001, uncorrected) in the SPM8 software and plotted on surface maps with the Surf Ice software—http://www.nitrc.org/projects/surfice/). Bars in the right side indicate *z* scores, ranging from *p* = 10^−3^ (*z*-score = 3.0) to *p* = 10^−4^ (*z*-score = 4.0).

Compared to healthy controls, group analysis on SPM from the whole cohort showed an extended pattern of rBGM reduction at frontoparietal areas, striatum, and thalamus, mostly contralateral to the affected body side (Figure 2A).

Patients with dysarthria were characterized by a predominant left-side hypometabolic pattern (Figure 2B), and more prominent rBGM reduction surviving correction for multiple comparisons at the cluster level at frontal regions, with a significant cluster at the left inferior frontal gyrus (opercular area) and left premotor cortex (Figure 2B), with additional features typical of CBS (inferior parietal cortex and striatum).

Conversely, patients without dysarthria showed bilateral rBGM reduction, with major clusters at the posterior cingulate, dorsolateral prefrontal cortex, posterior temporoparietal areas, striatum, and thalamus and no hemisphere predominance. See Figure 2 for details. Peak voxels of rBGM are shown in Supplementary Tables 1.1–1.3.

**Figure 3:**
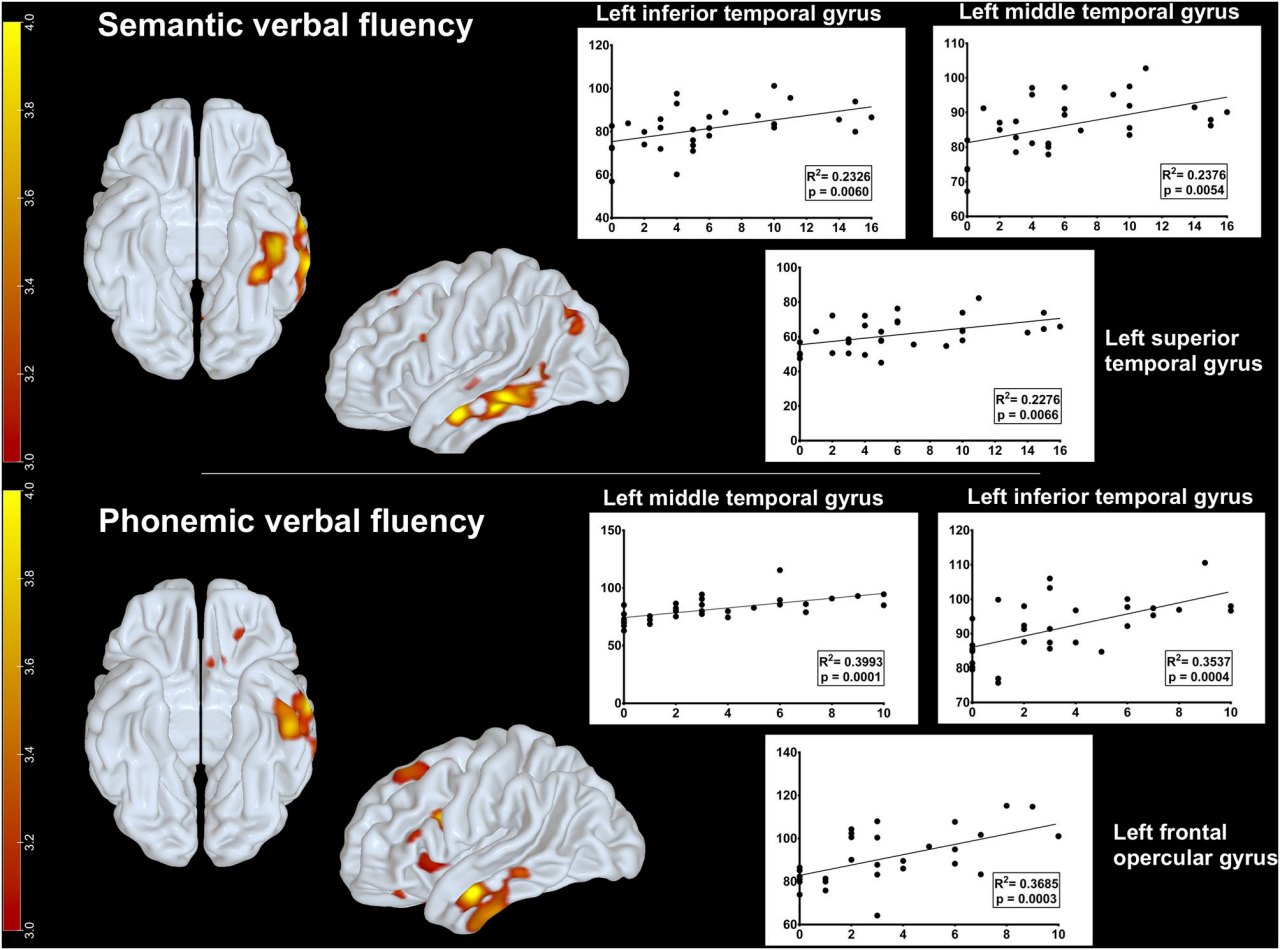
Metabolic correlations between brain regions and verbal fluency tasks. Upper row: positive correlation between glucose uptake on FDG-PET and semantic verbal fluency at the left inferior, middle, and superior temporal gyri. Lower row: positive correlation between glucose uptake on FDG-PET and phonemic fluency at the left frontal opercular gyrus and the inferior and middle temporal gyri. Parametric maps were generated with an unpaired *t*-test (threshold: *p* < 0.001, uncorrected) in the SPM8 software and plotted on surface maps with the Surf Ice software http://www.nitrc.org/projects/surfice/). Bars in the left side indicate *z* scores, ranging from *p* = 10^−3^ (*z*-score = 3.0) to *p* = 10^−4^ (*z*-score = 4.0).

Additionally, we investigated which brain regions on FDG-PET correlated with semantic and phonemic verbal fluency task performance. There was a positive correlation between rBGM and semantic verbal fluency at the left inferior (*p* = 0.006, *R*^2^ = 0.2326), middle (*p* = 0.0054, *R*^2^ = 0.2376), and superior temporal gyri (*p* = 0.0066, *R*^2^ = 0.2276) (Figure 3). Relative to the phonemic verbal fluency, we found a positive correlation between [^18^F]FDG uptake and letter P fluency at the left frontal opercular gyrus (*p* = 0.0003, *R*^2^ = 0.3685) and the inferior (*p* = 0.0004, *R*^2^ = 0.3537) and middle temporal gyri (*p* = 0.0001, *R*^2^ = 0.3993) (Figure 3).

### Brain Atrophy Patterns on VBM

Compared to healthy controls, the whole CBS cohort showed a widespread brain atrophy pattern with major clusters at the bilateral striatum, supplementary motor area (SMA), posterior cingulate cortex, and posterior temporoparietal areas mostly contralateral to the affected body side (Figure 2D).

In CBS patients with dysarthria, a major cluster of brain atrophy was found predominantly in the right inferior frontal gyrus and putamen, with other significant areas such as the left SMA, premotor cortex, and putamen (Figure 2E), whereas patients without dysarthria showed gray matter loss at posterior temporal and inferior parietal areas (Figure 2F). There was, however, no evident predominant left-side brain atrophy in patients with dysarthria. Peak voxels of VBM contrasts are shown in Supplementary Tables 1.5, 1.6.

## Discussion

This prospective cross-sectional study described speech and language profiles in a cohort of 31 CBS patients assessed with a specific ligand for brain amyloid deposition. Our goal was to distinguish language and motor speech deficits related to amyloid-positive and amyloid-negative CBS patients and explore its brain metabolic and structural signatures through a multimodal imaging approach.

As our main findings, CBS patients with negative amyloid-PET presented dysarthria significantly more often than did patients with positive amyloid deposition. Additionally, quantitative FDG-PET and MRI group analyses showed differential hypometabolic and brain atrophy patterns in patients with and without dysarthria compared to healthy controls. Namely, CBS patients with dysarthria had a left-sided hypometabolism and bilateral brain atrophy pattern mainly at the opercular frontal region, premotor cortex, and SMA (see Figures 2B,E).

Motor speech production deficits such as dysarthria and AOS have been previously linked to CBS with underlying 4R tauopathy pathologies, such as CBD or PSP (9, 21, 22, 45). Dysarthria is considered a CBD and PSP frequent symptom from its first descriptions (2, 46) until their latest criteria (1, 47). Our results are in line with these previous studies. Furthermore, the regions with significant clusters of brain atrophy at MRI-based VBM in CBS patients with dysarthria were previously described to be anatomically involved in the motor speech production network (48). It is worth mentioning that AOS was also more commonly found in CBS-A– patients, although not achieving statistical significance.

In this cohort, aphasia was one of the most prominent cognitive impairments, present in 67.7% of the cases, second only to apraxia (96.8%). We identified a broad spectrum of the linguistic profile, ranging from the nfv-PPA phenotype to lexical-semantic deficits. The CBS-A+ group showed aphasia (77%) more often than did the CBS-A– group (61%) but without a statistically significant difference. Our data are congruent with a previous systematic literature review (1) and a recent clinicopathologic study (49) which demonstrated that aphasia occurred in more than 50% of CBS cases during the disease course.

Likewise, a prior retrospective study with a large cohort suggested that CBS consisted of a primarily language-motor disease with a predominant phenotype of mixed aphasia, thereby being the main cognitive feature (15). Our findings, along with these reports, strengthen the concept that language impairment, initially underscored in CBS, should be considered a cognitive hallmark of the disease.

In a previous study from our group with the same cohort, differences in rBGM in CBS patients were investigated according to amyloid imaging status. A quantitative group analysis showed hypometabolism comprising the posterior temporoparietal areas, mainly contralateral to the most affected side, as the areas with the most consistent hypometabolism in amyloid-positive CBS patients. Amyloid-negative patients, conversely, showed more heterogeneous metabolic patterns and disclosed areas of rBGM reduction at the thalamus and SMA (36).

In this present study, patients with dysarthria showed clusters of rBGM reduction at frontal regions, mainly at the left opercular region, premotor cortex, and SMA, corroborating a previous finding that patients with nfv-PPA who later evolved into CBS shared a left-sided pattern involving the inferior frontal gyrus and the supplementary motor cortex (25). In this article, the authors provide further evidence that the topography of brain hypometabolism could reflect dysfunctional signatures of different language deficits. Although most patients with dysarthria in our cohort did not fulfill the criteria for nfv-PPA, they might pertain to the same language dysfunctional spectrum commonly found in the group with CBS with underlying 4R tauopathies.

It is acknowledged that the wide variety of aphasic syndromes in CBS probably derive from the diversity of underlying pathologies or is a function of the stage when the clinical assessment occurs (23). A logopenic-like aphasia phenotype, with poor sentence repetition, anomia, and word retrieval problems, has been associated with an underlying AD pathology in a previous clinicopathological series (49) and a study using amyloid-PET (26). However, we could not replicate these prior findings of logopenic-PPA phenotype in the CBS-A+ group from our cohort. Meanwhile, patients in the CBS-A+ group presented worse cognitive performances at MMSE and ACE-R attention, memory, and visuospatial subscores, findings earlier highlighted in *postmortem* (7, 45) and *in vivo* biomarkers-based (15, 36) research works. We hypothesize that the advanced functional stage and compromised cognition detected in the CBS-A+ group may have prevented us from obtaining this observation. Otherwise, one additional possibility is that the language profiles are too heterogeneous in CBS and it is often not possible to delineate a unique pattern.

The majority of our patients demonstrated phonemic and semantic verbal fluency impairment. It is recognized that verbal fluency performance relates not only to language dysfunction but also to other cognitive domains such as executive function and attention, reflecting initiation and processing speed. Notably, the CBS-A– group tended to show a more compromised phonemic verbal fluency, while the CBS-A+ group had a worse semantic verbal fluency performance, even though it did not reach statistical significance. Most studies have reported reduced word fluency in CBS patients (15, 50), especially concerning phonemic fluency. In line with our findings, a previous research work revealed significant impairment in the CBS-A– group regarding the phonemic verbal fluency task compared to the CBS-A+ group (51). As we consider that cases from the CBS-A– group probably encompass CBD and PSP pathologies and adding the fact that PSP studies have shown even more impairment related to phonemic verbal fluency, we might thus find a rationale to this pattern (23, 27).

Additionally, we assessed neural correlates from verbal fluency performance in CBS patients, a matter that has not been extensively investigated (23). Semantic verbal fluency correlated positively with glucose metabolism in the left superior, middle, and inferior temporal gyri, whereas phonemic verbal fluency correlated with metabolism in the left frontal areas, mainly at the left inferior frontal gyrus, and with left temporal areas, comprising the middle and inferior temporal gyri (see Figure 3). These findings are consistent with data from functional imaging in healthy adults (52).

The main limitation of our study was the lack of histopathological data or other pathology *in vivo* tracers, such as tau-PET. In its absence, we could not correctly distinguish the language profile concerning underlying pathologies in the group with negative amyloid deposition or investigate the influence of comorbid pathologies in language dysfunction. In a previous study, patients with nfv-PPA and underlying PSP pathology showed more dysarthria than those with nfv-PPA with CBD pathology (24). Therefore, there is a possibility that our patients in the CBS-A– group with dysarthria had more underlying PSP pathology than CBD. Positive aspects are a relatively significant number of CBS patients from a unique center, with standardized neurological, cognitive, and speech–language assessment, studied with multimodal imaging from the same protocols with blinded analysis for the diagnosis, including a specific ligand for amyloid pathology.

Finally, we could depict two groups (CBS-A+ and CBS-A–) with distinct motor speech features and cognitive performances, but without a clear difference concerning language profile. Our results shed light on dysarthria as an aspect related to the CBS-A– variant, and thus, it might be a helpful clinical clue suggesting the underlying CBS pathology. Also, we found metabolic and structural signatures related to the presence of dysarthria that provide insights into the motor speech production networks. Further longitudinal studies with larger samples are warranted to encompass the diversity of language impairment in distinct stages of CBS disease progression.

## Data Availability Statement

The original contributions presented in the study are included in the article/Supplementary Material, further inquiries can be directed to the corresponding author/s.

## Ethics Statement

The studies involving human participants were reviewed and approved by Ethical committee of University of São Paulo under protocol number 2.046.113. The patients/participants provided their written informed consent to participate in this study.

## Author Contributions

JP, IA, and AC: designed and conceptualized the study and data collection and drafted the manuscript for intellectual content. MO: statistics, analyzed and interpreted the data, and revised the manuscript for intellectual content. AS-N, CG, and CO: data collection. MS, ER, RN, CB, and SB: revised the manuscript for intellectual content. All authors contributed to the article and approved the submitted version.

## Conflict of Interest

The authors declare that the research was conducted in the absence of any commercial or financial relationships that could be construed as a potential conflict of interest.

## Publisher's Note

All claims expressed in this article are solely those of the authors and do not necessarily represent those of their affiliated organizations, or those of the publisher, the editors and the reviewers. Any product that may be evaluated in this article, or claim that may be made by its manufacturer, is not guaranteed or endorsed by the publisher.
