# Supplementary material for: Metabolic and Structural Signatures of Speech and Language Impairment in Corticobasal Syndrome: A Multimodal PET/MRI Study
Source: Front Neurol. 2021 Aug 30;12:702052. doi: 10.3389/fneur.2021.702052 (PMC8435851; doi:10.3389/fneur.2021.702052)
Supplement: Supplementary file 1 [file Table_1.docx]

Supplementary Material

# Supplementary tables

## Demographical and neuropsychological evaluation of healthy control participants.

| Number of participants | 30 |
| --- | --- |
| Gender (male/female) | 4/26 |
| Age at assessment, y | 67.3 (65.8 – 68.8) |
| Education, y | 13.8 (12.2 – 15.5) |
| MMSE | 28.9 (28.5 – 29.3) |
| MoCA | 25.1 (24.4 – 25.9) |
| Delayed recall (BCSB) | 8.3 (8.0 – 8.7) |
| Digits backward | 5.35 (4.7 – 6.0) |
| Semantic fluency (animals) | 19.0 (16.6 – 21.2) |
| Phonemic fluency (letter P) | 14.5 (12.7 – 16.3) |

**Legends:** Data expressed as median (interquartile range) or number (frequency). Abbreviations: MMSE, Mini-mental State Examination; MoCA: Montreal cognitive assessment; BCSB, Brief Cognitive Screening Battery; y, years.

## Areas of regional brain glucose metabolism (rGBM) reduction in corticobasal syndrome subjects compared to healthy controls.

| Area | cluster-level | | | | peak-level | | | | MNI coordinates | | |
| --- | --- | --- | --- | --- | --- | --- | --- | --- | --- | --- | --- |
|  | *p*_FWE_ | *p*_FDR_ | k | *p*_uncorr_ | *p*_FWE_ | *p*_FDR_ | Z_(E)_ | *p*_uncorr_ | mm | mm | mm |
| *Middle frontal gyrus (contralateral to most affected side)* | **0.000** | **0.000** | **13273** | **0.000** | **0.000** | **0.000** | **inf** | **0.000** | **44** | **10** | **56** |
| *Supplementary motor area (contralateral to most affected side)* |  |  |  |  | 0.000 | 0.000 | inf | 0.000 | 32 | -5 | 56 |
| *Premotor cortex (contralateral to most affected side)* |  |  |  |  | 0.000 | 0.000 | 7.36 | 0.000 | 48 | 5 | 38 |
| *Posterior cingulate cortex (contralateral to most affected side)* | **0.000** | **0.000** | **1329** | **0.000** | **0.000** | **0.000** | **7.33** | **0.000** | **4** | **-24** | **32** |
| *Ventral cingulate area (contralateral to most affected side)* |  |  |  |  | 0.000 | 0.000 | 7.24 | 0.000 | 5 | 10 | 30 |
| *Thalamus (contralateral to most affected side)* | **0.000** | **0.000** | **429** | **0.000** | **0.000** | **0.000** | **7.02** | **<0.001** | **12** | **-18** | **12** |
| *Supramarginal gyrus (contralateral to most affected side)* | **0.000** | **0.009** | **134** | **0.002** | **0.002** | **0.048** | **5.34** | **0.000** | **50** | **-40** | **-34** |
| *Caudate (ipsilateral to most affected side)* | **0.000** | **0.017** | **109** | **0.004** | **0.000** | **0.000** | **6.78** | **0.000** | **-12** | **8** | **10** |

MNI: Montreal Neurological Institute; FDG: [^18^F]fluorodeoxyglucose; FWE: Familywise Error; FDR: False Discovery Rate; *p*_FWE_: *p* value corrected for multiple comparisons using FWE method; *p*_FDR_: *p* value corrected for multiple comparisons using FDR method; *p*_uncorr_: *p* value uncorrected for multiple comparisons; k_E_: cluster size (in number of voxels), Z_(E)_: Z-score, GM: gray matter.

## Areas of regional brain glucose metabolism (rGBM) reduction in corticobasal syndrome patients with dysarthria compared to healthy controls.

| Area | cluster-level | | | | peak-level | | | | MNI coordinates | | |
| --- | --- | --- | --- | --- | --- | --- | --- | --- | --- | --- | --- |
|  | *p*_FWE_ | *p*_FDR_ | k | *p*_uncorr_ | *p*_FWE_ | *p*_FDR_ | Z_(E)_ | *p*_uncorr_ | mm | mm | mm |
| *Left Premotor cortex* | **0.000** | **0.000** | **2189** | **0.000** | **0.018** | **0.018** | **4.95** | **0.000** | **-36** | **8** | **58** |
| *Left inferior frontal/opercular gyrus* |  |  |  |  | 0.059 | 0.047 | 4.66 | 0.000 | -50 | 6 | 24 |
| *Left temporal pole* |  |  |  |  | 0.059 | 0.047 | 4.66 | 0.000 | -50 | 18 | -20 |
| *Left supramarginal gyrus* | **0.000** | **0.000** | **1208** | **0.000** | **0.008** | **0.014** | **5.12** | **0.000** | **-58** | **-30** | **46** |
| *Left angular gyrus* |  |  |  |  | 0.173 | 0.068 | 4.37 | 0.000 | -42 | -50 | 44 |
| *Left caudate* | **0.001** | **0.001** | **587** | **0.000** | **0.002** | **0.010** | **5.45** | **0.000** | **-14** | **2** | **16** |
| *Left thalamus* |  |  |  |  | 0.096 | 0.052 | 4.53 | 0.000 | -8 | -16 | 12 |
| *Left supplementary motor area* | **0.349** | **0.142** | **119** | **0.045** | **0.236** | **0.083** | **4.27** | **0.000** | **-6** | **6** | **72** |
| *Right dorsomedial prefrontal cortex* | **0.000** | **0.000** | **2350** | **0.000** | **0.079** | **0.052** | **4.58** | **0.000** | **44** | **10** | **56** |
| *Right anterior cingulate* |  |  |  |  | 0.141 | 0.062 | 4.43 | 0.000 | 4 | 16 | 26 |
| *Right caudate* | **0.339** | **0.142** | **121** | **0.043** | **0.009** | **0.014** | **5.11** | **0.000** | **16** | **8** | **10** |

MNI: Montreal Neurological Institute; FDG: [^18^F]fluorodeoxyglucose; FWE: Familywise Error; FDR: False Discovery Rate; *p*_FWE_: *p* value corrected for multiple comparisons using FWE method; *p*_FDR_: *p* value corrected for multiple comparisons using FDR method; *p*_uncorr_: *p* value uncorrected for multiple comparisons; k_E_: cluster size (in number of voxels), Z_(E)_: Z-score, GM: gray matter.

## Areas of regional brain glucose metabolism (rGBM) reduction in corticobasal syndrome patients without dysarthria compared to healthy controls.

| Area | cluster-level | | | | peak-level | | | | MNI coordinates | | |
| --- | --- | --- | --- | --- | --- | --- | --- | --- | --- | --- | --- |
|  | *p*_FWE_ | *p*_FDR_ | k | *p*_uncorr_ | *p*_FWE_ | *p*_FDR_ | Z_(E)_ | *p*_uncorr_ | mm | mm | mm |
| *Right ventral posterior cingulate cortex* | **0.000** | **0.000** | **1246** | **0.000** | **0.000** | **0.000** | **7.43** | **0.000** | **0** | **-24** | **32** |
| *Right dorsal posterior cingulate* |  |  |  |  | 0.000 | 0.000 | 6.76 | 0.000 | 14 | -28 | 40 |
| *Right dorsolateral Prefrontal cortex* | **0.000** | **0.000** | **2329** | **0.000** | **0.000** | **0.000** | **6.90** | **0.000** | **40** | **34** | **38** |
| *Right superior frontal gyrus* |  |  |  |  | 0.000 | 0.003 | 6.13 | 0.000 | 40 | 20 | 52 |
| *Right supramarginal gyrus* | **0.000** | **0.000** | **2026** | **0.000** | **0.000** | **0.001** | **6.59** | **0.000** | **58** | **-44** | **48** |
| *Right angular gyrus* |  |  |  |  | 0.000 | 0.006 | 5.92 | 0.000 | 50 | -58 | 48 |
| *Right superior temporal gyrus* |  |  |  |  | 0.001 | 0.026 | 5.56 | 0.000 | 56 | -36 | 20 |
| *Left supramarginal gyrus* | **0.000** | **0.000** | **2483** | **0.000** | **0.000** | **0.001** | **6.46** | **0.000** | **-58** | **-38** | **46** |
| *Left angular gyrus* |  |  |  |  | 0.000 | 0.002 | 6.25 | 0.000 | -46 | -66 | 48 |
| *Left dorsolateral Prefrontal cortex* | **0.000** | **0.000** | **616** | **0.000** | **0.000** | **0.003** | **6.21** | **0.000** | **-36** | **38** | **34** |
| *Left superior frontal gyrus* |  |  |  |  | 0.004 | 0.114 | 5.19 | 0.000 | -44 | 18 | 44 |
| *Left Caudate* | **0.000** | **0.000** | **201** | **0.000** | **0.000** | **0.001** | **6.52** | **0.000** | **-14** | **8** | **10** |
| *Left Thalamus* |  |  |  |  | 0.000 | 0.005 | 5.99 | 0.000 | -8 | -14 | 12 |

MNI: Montreal Neurological Institute; FDG: [^18^F]fluorodeoxyglucose; FWE: Familywise Error; FDR: False Discovery Rate; *p*_FWE_: *p* value corrected for multiple comparisons using FWE method; *p*_FDR_: *p* value corrected for multiple comparisons using FDR method; *p*_uncorr_: *p* value uncorrected for multiple comparisons; k_E_: cluster size (in number of voxels), Z_(E)_: Z-score, GM: gray matter.

## Areas of brain atrophy in corticobasal syndrome subjects compared to healthy controls.

| Area | cluster-level | | | | peak-level | | | | MNI coordinates | | |
| --- | --- | --- | --- | --- | --- | --- | --- | --- | --- | --- | --- |
|  | *p*_FWE_ | *p*_FDR_ | k | *p*_uncorr_ | *p*_FWE_ | *p*_FDR_ | Z_(E)_ | *p*_uncorr_ | mm | mm | mm |
| *Putamen (contralateral to most affected side)* | **0.000** | **0.000** | **1331** | **0.000** | **0.000** | **0.000** | **7.21** | **0.000** | **26** | **10** | **-10** |
| *Insula (contralateral to most affected side)* |  |  |  |  | 0.004 | 0.112 | 5.20 | 0.000 | 42 | 10 | 2 |
| *Posterior cingulate cortex (contralateral to most affected side)* | **0.000** | **0.000** | **294** | **0.000** | **0.000** | **0.003** | **5.98** | **0.000** | **6** | **-14** | **48** |
| *Supplementary motor area (contralateral to most affected side)* |  |  |  |  | 0.000 | 0.016 | 5.65 | 0.000 | 4 | -10 | 46 |
| *Putamen (ipsilateral to most affected side)* | **0.000** | **0.000** | **1145** | **0.000** | **0.000** | **0.000** | **7.47** | **0.000** | **-24** | **10** | **-6** |

MNI: Montreal Neurological Institute; FDG: [^18^F]fluorodeoxyglucose; FWE: Familywise Error; FDR: False Discovery Rate; *p*_FWE_: *p* value corrected for multiple comparisons using FWE method; *p*_FDR_: *p* value corrected for multiple comparisons using FDR method; *p*_uncorr_: *p* value uncorrected for multiple comparisons; k_E_: cluster size (in number of voxels), Z_(E)_: Z-score, GM: gray matter.

## Areas of brain atrophy in corticobasal syndrome patients with dysarthria compared to healthy controls.

| Area | cluster-level | | | | peak-level | | | | MNI coordinates | | |
| --- | --- | --- | --- | --- | --- | --- | --- | --- | --- | --- | --- |
|  | *p*_FWE_ | *p*_FDR_ | k | *p*_uncorr_ | *p*_FWE_ | *p*_FDR_ | Z_(E)_ | *p*_uncorr_ | mm | mm | mm |
| *Right Putamen* | **0.000** | **0.000** | **1794** | **0.000** | **0.000** | **0.000** | **6.05** | **0.000** | **26** | **12** | **-10** |
| *Right inferior frontal/opercular gyrus* |  |  |  |  | 0.821 | 0.509 | 3.70 | 0.000 | 40 | 10 | 4 |
| *Left Putamen* | **0.000** | **0.001** | **753** | **0.000** | **0.057** | **0.076** | **4.65** | **0.000** | **-16** | **10** | **-8** |
| *Left supplementary motor area* | **0.010** | **0.007** | **435** | **0.001** | **0.314** | **0.253** | **4.16** | **0.000** | **-4** | **-18** | **52** |
| *Left Premotor cortex* |  |  |  |  | 0.997 | 0.791 | 3.27 | 0.001 | -4 | 6 | 44 |
| *Right middle temporal gyrus* | **0.017** | **0.010** | **379** | **0.002** | **0.194** | **0.209** | **4.32** | **0.000** | **58** | **-32** | **-2** |
| *Right angular gyrus* |  |  |  |  | 0.993 | 0.744 | 3.34 | 0.000 | 60 | -50 | 18 |
| *Right middle cingulate cortex* | **0.024** | **0.011** | **351** | **0.003** | **0.707** | **0.509** | **3.81** | **0.000** | **6** | **24** | **32** |
| *Right anterior cingulate cortex* |  |  |  |  | 0.97 | 0.678 | 3.46 | 0.000 | 8 | 2 | 40 |
| *Right Cerebellum* | **0.053** | **0.020** | **281** | **0.006** | **0.402** | **0.253** | **4.07** | **0.000** | **8** | **-56** | **-52** |
| *Left Cerebellum* | **0.123** | **0.042** | **211** | **0.015** | **0.841** | **0.509** | **3.68** | **0.000** | **-18** | **-70** | **-56** |
| *Left Precentral* | **0.421** | **0.151** | **114** | **0.061** | **0.952** | **0.678** | **3.51** | **0.000** | **-36** | **-10** | **48** |

MNI: Montreal Neurological Institute; FDG: [^18^F]fluorodeoxyglucose; FWE: Familywise Error; FDR: False Discovery Rate; *p*_FWE_: *p* value corrected for multiple comparisons using FWE method; *p*_FDR_: *p* value corrected for multiple comparisons using FDR method; *p*_uncorr_: *p* value uncorrected for multiple comparisons; k_E_: cluster size (in number of voxels), Z_(E)_: Z-score, GM: gray matter.

## Areas of brain atrophy in corticobasal syndrome patients without dysarthria compared to healthy controls.

| Area | cluster-level | | | | peak-level | | | | MNI coordinates | | |
| --- | --- | --- | --- | --- | --- | --- | --- | --- | --- | --- | --- |
|  | *p*_FWE_ | *p*_FDR_ | k | *p*_uncorr_ | *p*_FWE_ | *p*_FDR_ | Z_(E)_ | *p*_uncorr_ | mm | mm | mm |
| *Right Putamen* | **0.000** | **0.000** | **1327** | **0.000** | **0.000** | **0.000** | **7.18** | **0.000** | **28** | **8** | **0** |
| *Right Insula* |  |  |  |  | 0.001 | 0.041 | 5.50 | 0.000 | 42 | 10 | 2 |
| *Left Striatum* | **0.000** | **0.000** | **664** | **0.000** | **0.000** | **0.000** | **6.55** | **0.000** | **-22** | **10** | **-6** |
| *Right Anterior Cingulate* | **0.000** | **0.001** | **118** | **0.001** | **0.000** | **0.003** | **6.10** | **0.000** | **10** | **-18** | **44** |
| *Right middle temporal gyrus* | **0.000** | **0.001** | **125** | **0.001** | **0.003** | **0.079** | **5.29** | **0.000** | **62** | **-32** | **0** |
| *Right superior temporal gyrus* |  |  |  |  | 0.009 | 0.190 | 5.07 | 0.000 | 62 | -40 | 6 |

MNI: Montreal Neurological Institute; FDG: [^18^F]fluorodeoxyglucose; FWE: Familywise Error; FDR: False Discovery Rate; *p*_FWE_: *p* value corrected for multiple comparisons using FWE method; *p*_FDR_: *p* value corrected for multiple comparisons using FDR method; *p*_uncorr_: *p* value uncorrected for multiple comparisons; k_E_: cluster size (in number of voxels), Z_(E)_: Z-score, GM: gray matter.
