# Supplementary material for: Metabolic and Structural Signatures of Speech and Language Impairment in Corticobasal Syndrome: A Multimodal PET/MRI Study
Source: Front Neurol. 2021 Aug 30;12:702052. doi: 10.3389/fneur.2021.702052 (PMC8435851; doi:10.3389/fneur.2021.702052)
Supplement: Supplementary file 2 [file Data_Sheet_1.docx]

Excessive White Matter Hyperintensity Increases Susceptibility to Poor Functional Outcomes after Acute Ischemic Stroke

# Supplementary

**Table S1.** Population clinical characteristics of AIS patients including non-survivors (mRS=6) used for the analysis from the MRI-GENIE study, and the low, expected and excessive uWMH burden groups.

|  | Total (n=924) | Low (n=139) | Expected (n=631) | Excessive (n=154) |
| --- | --- | --- | --- | --- |
| Age, avg (std) | 64.3 (15.1) | 61.8 (14.4) | 64.8 (15.5) | 64.4 (13.8) |
| Sex, n (%) | 397 (43.0%) | 60 (43.2%) | 266 (42.2%) | 71 (46.1%) |
| HTN, n (%) | 573 (62.0%) | 87 (62.6%) | 385 (61.0%) | 101 (65.6%) |
| DM, n (%) | 190 (20.6%) | 22 (15.8%) | 142 (22.5%) | 26 (16.9%) |
| AF, n (%) | 157 (17.0%) | 18 (12.9%) | 114 (18.1%) | 25 (16.2%) |
| CAD, n (%) | 155 (16.8%) | 11 (7.9%) | 120 (19.0%) | 24 (15.6%) |
| SMK, n (%) | 488 (52.8%) | 68 (48.9%) | 345 (54.7%) | 75 (48.7%) |
| PS, n (%) | 89 (9.6%) | 13 (9.4%) | 62 (9.8%) | 14 (9.1%) |
| NIHSS, median (IQR) | 3 (5) | 3 (4) | 3 (6) | 3 (5) |
| uWMH, avg (std) | 0 (1.1) | -1.8 (0.6) | 0.0 (0.6) | 1.6 (0.4) |

**Table S2.**The regression coefficients of individual univariate and multiple log-linear regression analyses that modeled the associations of age, sex, VRFs, and NIHSS with WMHv for the entire population including non-survivors (mRS=6).

| Y=WMHv | Age | Sex | HTN | AF | CAD | DM | SMK | PS | NIHSS | $\beta_{0}$ |
| --- | --- | --- | --- | --- | --- | --- | --- | --- | --- | --- |
| Regression Coefficients | 0.06 | -0.19 | 0.33 | -0.03 | -0.1 | 0.26 | 0.29 | 0.37 | 0.01 | -9.78 |
| $p$ | **<0.01** | **0.02** | **<0.01** | 0.75 | 0.35 | **<0.01** | **<0.01** | **<0.01** | 0.1 | **<0.01** |

**Table S3.**The odds ratios of entire patients including non-survivors (mRS=6) who achieved full functional independence (mRS Excellent: mRS scores 0-1) of the excessiveuWMH burden groups to the low (Excessive/Low) and expected (Excessive/Expected) groups, and of the expected group to the low (Expected/Low) group.

| Population | Excessive/Low | Excessive/Expected | Expected/Low |
| --- | --- | --- | --- |
| OR | 0.4 | 0.65 | 0.62 |
| p, 95% CI | **< 0.01**, [0.25, 0.64] | **0.02**, [0.45, 0.92] | **0.01**, [0.42, 0.9] |

**Table S4.** Population clinical characteristics of all AIS patients including non-survivors (mRS=6) in mild acute stroke severity group (Mild, $NIHSS<7$) and severe stroke severity group (Severe, $NIHSS\geq7$).

|  | Mild (n=681) | Severe (n=243) |
| --- | --- | --- |
| Age, avg (std) | 63.7 (15.2) | 65.9 (14.6) |
| Sex, n (%) | 277 (40.7%) | 120 (49.4%) |
| HTN, n (%) | 423 (62.1%) | 150 (61.7%) |
| DM, n (%) | 138 (20.3%) | 52 (21.4%) |
| AF, n (%) | 93 (13.7%) | 64 (26.3%) |
| CAD, n (%) | 104 (15.3%) | 51 (21.0%) |
| SMK, n (%) | 373 (54.8%) | 115 (47.3%) |
| PS, n (%) | 65 (9.5%) | 24 (9.9%) |
| NIHSS, median (IQR) | 2 (3) | 11 (8) |
| uWMH, avg (std) | 0.0 (1.1) | 0.0 (1.1) |

**Table S5.** Acute Stroke Severity Subgroup Analysis for entire population including non-survivors (mRS=6).The odds ratios of patients who achieved full functional independence (mRS Excellent: mRS scores 0-1) of the excessive uWMH burden groups to the low (Excessive/Low) and expected (Excessive/Expected) groups, and of the expected group to the low (Expected/Low) group in the mild acute stroke severity group (Mild, $NIHSS<7$) and the severe acute stroke severity group (Severe,$NIHSS\geq7$).

| Acute Stroke Severity Group | | Excessive/Low | Excessive/Expected | Expected/Low |
| --- | --- | --- | --- | --- |
| Mild (n=681) | OR | 0.44 | 0.59 | 0.75 |
|  | p, 95% CI | **< 0.01**, [0.25, 0.77] | **0.01**, [0.39, 0.89] | 0.21, [0.47, 1.18] |
| Severe (n=243) | OR | 0.24 | 0.65 | 0.37 |
|  | p, 95% CI | **< 0.01**, [0.08, 0.7] | 0.34, [0.27, 1.58] | **0.01**, [0.17, 0.8] |
